# Gravitational effects of scene information in object localization

**DOI:** 10.1038/s41598-021-91006-8

**Published:** 2021-06-01

**Authors:** Anna Kosovicheva, Peter J. Bex

**Affiliations:** 1 Department of Psychology, University of Toronto Mississauga, 3359 Mississauga Road, Mississauga, ON L5L 1C6 Canada; 2 Department of Psychology, Northeastern University, 125 Nightingale Hall, 360 Huntington Ave., Boston, MA 02115 USA

**Keywords:** Visual system, Object vision, Human behaviour, Perception

## Abstract

We effortlessly interact with objects in our environment, but how do we know where something is? An object’s apparent position does not simply correspond to its retinotopic location but is influenced by its surrounding context. In the natural environment, this context is highly complex, and little is known about how visual information in a scene influences the apparent location of the objects within it. We measured the influence of local image statistics (luminance, edges, object boundaries, and saliency) on the reported location of a brief target superimposed on images of natural scenes. For each image statistic, we calculated the difference between the image value at the physical center of the target and the value at its reported center, using observers’ cursor responses, and averaged the resulting values across all trials. To isolate image-specific effects, difference scores were compared to a randomly-permuted null distribution that accounted for any response biases. The observed difference scores indicated that responses were significantly biased toward darker regions, luminance edges, object boundaries, and areas of high saliency, with relatively low shared variance among these measures. In addition, we show that the same image statistics were associated with observers’ saccade errors, despite large differences in response time, and that some effects persisted when high-level scene processing was disrupted by 180° rotations and color negatives of the originals. Together, these results provide evidence for landmark effects within natural images, in which feature location reports are pulled toward low- and high-level informative content in the scene.

## Introduction

To successfully interact with our environment, we must accurately perceive the locations of objects around us. This is central to our ability to perform a variety of tasks, such as catching a baseball or reaching for a pen. Although position information is coded retinotopically at multiple stages of visual processing, perceived location can be influenced by many other factors beyond retinal location. Factors such as eye movements^1^, motion^2–4^, stimulus history^5,6^, visual attention^7^, and individual differences^8^ can influence position assignment. Many of these phenomena may reflect mechanisms that facilitate our interaction with the environment, including maintenance of stability across eye movements^1^, and over time^9^.

An important contribution to position representations is the influence of nearby visual features on localization. Effects of this type include shifts in the perceived location or orientation of a static object based on the appearance of a surrounding frame^10,11^. Similarly, when observers report the location of a previously seen target, responses are often pulled toward other stationary landmarks or spatial references^12–17^. Although these effects may generally reflect remembered—rather than perceived—location, other studies have reported illusions in which the apparent position of a target is shifted toward nearby references or anchors^18–20^. These effects have typically been studied with simple stimulus configurations on uniform backgrounds in which patterns of error in reproducing the position of a single target are measured with or without static references. Despite the relevance for action in everyday tasks, much less is known about how these factors operate in more natural settings, or how local image features contribute to position assignment within natural scenes.

When an observer is shown a briefly flashed target within a scene, how does the information in the scene affect where they localize it? Although observers could, conceivably, ignore scene information, either localizing the briefly presented object accurately or with some error that is random with respect to the scene, this is unlikely given this previous literature. One possibility is that the types of landmark effects observed in more simple configurations may appear in more naturalistic settings and pull the reported object locations toward nearby references. Additionally, localization biases could reflect priors for expected object location, based on the spatial distribution of objects learned from experience with natural settings. For example, objects are unlikely to be suspended in midair or far from other objects, and this knowledge might bias an observer’s report of target positions in natural settings.

**Figure 1:**
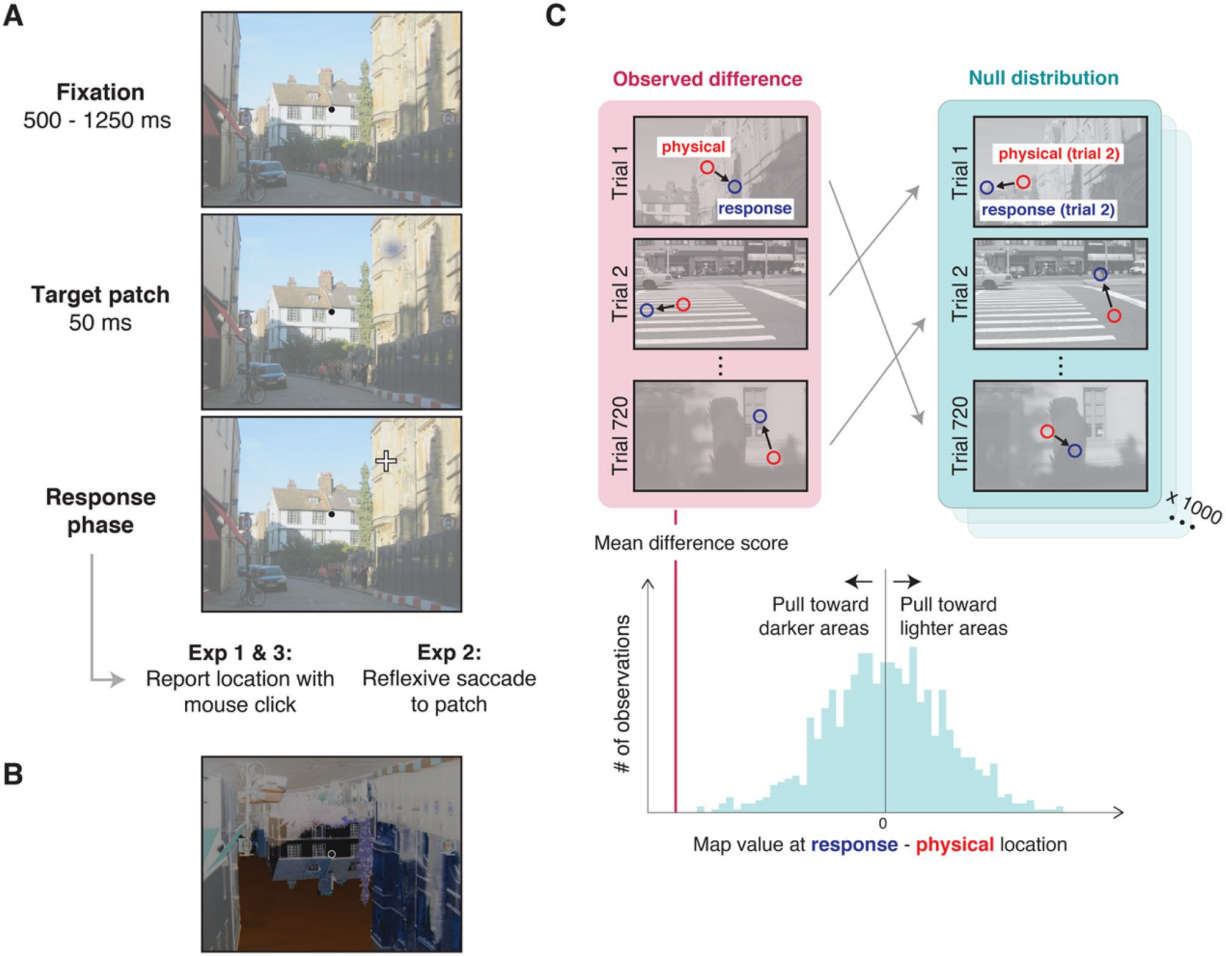
(**A**) On each trial, observers continuously fixated a dot at the center of the display for a random interval between 500 and 1250 ms. A brief (50 ms) Gaussian target patch was randomly presented at one of 72 possible peripheral locations (3 eccentricities × 24 angular locations; shown here in the upper-right portion of the image). Observers then either reported its location by moving a crosshair controlled by a mouse while maintaining fixation (Experiments 1 and 3), or made a reflexive saccade to the target (Experiment 2). (**B**) The images used in Experiment 3 were color negatives of the original image, and flipped upside down. (**C**) To measure the influence of different image features on localization errors, we calculated difference scores from the image value at the physical center of the patch location (“physical”; red) and the location reported by the observer (“response”; blue, hypothetical example shown with luminance maps). In the example, negative values indicate that responses are pulled toward darker areas in the image, and positive values indicate that responses are pulled toward lighter areas. To isolate image-specific effects from other potential reporting biases, the observed mean difference score was compared to values in a null distribution, which was calculated by randomly shuffling the physical and response coordinates in relation to other images. In the example, the images in the right column are in the same order as the original trials, but the coordinates are taken from different trials (randomly shuffled on each iteration), indicated by the gray arrows. For example, the coordinates from trial 2 were used to calculate a difference score for the image in trial 1. A mean difference score was calculated from all shuffled trials, and this procedure was repeated for 1000 iterations to produce a null distribution of difference scores.

To identify the ‘landmarks’ that influence position judgments under uncertainty within natural images, we measured the influence of local image statistics on target localization, similar to approaches to investigate detection and identification within natural scene images^21–23^. On each trial, observers reported the perceived location of a briefly presented Gaussian patch by adjusting the position of a cursor to match its apparent location (Fig. 1A). We analyzed observers’ errors in relation to meaningful features or statistics within the images, which although not fully independent, have relatively low shared variance (see *Results*), and therefore have the potential to capture different information in these images.

We adopted an exploratory approach, using a number of features that have been previously shown to influence detection or identification of elements^21–23^ as well as attention or eye movements^24,25^ within natural scenes. This included lower-level features such as luminance and luminance-defined edge information (i.e., distance from nearest edge). We also included an edge density measure, which captures information about locations where there is a high density of luminance boundaries (e.g., closely-spaced textures), and has been shown to influence detection of targets within natural scenes^21^. In addition, we included higher-level information about the outlines of objects in the image by measuring errors in relation to annotated object outlines^26^ in the images. Finally, we included a measure of saliency^27^, as a way to identify possible attentional landmarks in the images, as attention has been previously shown to influence spatial position judgments under more simple configurations^7^.

In Experiment 1, we established the image-specific influences of each of these features on observers’ localization errors, demonstrating that observers’ responses were significantly biased toward darker regions, luminance edges, object boundaries, and areas of high saliency. To demonstrate that these effects were not specific to cursor responses, Experiment 2 reproduced this result by showing that similar features contributed to observers’ saccade errors. Finally, in Experiment 3 (Fig. 1B), we used inverted and color-negative images to demonstrate that a subset of these gravitational biases persist when normal scene processing is disrupted, separating lower-level and higher-level influences of these features on position representations.

## Methods

### Participants

We collected data from ten participants in Experiment 1 (6 female, 4 male, ages 18–37), twelve in Experiment 2 (9 female, 3 male, 18–33), and twelve in Experiment 3 (8 female, 4 male, ages 18–30). One of the authors participated in Experiments 1 and 2. All participants reported normal or corrected-to-normal vision, and all except the author were naïve to the purpose of the study. The experiments and their respective analyses were pre-registered on the Open Science Framework (https://osf.io/d7qrv/registrations). Procedures were approved by the Institutional Review Board at Northeastern University and the experiments were carried out in accordance with the relevant regulations regarding human subjects research. All observers gave informed consent prior to participating in the experiment.

### Eye tracking

Eye movements were recorded with an Eyelink 1000 desktop mounted infrared eye tracker (SR Research Ltd., Ottawa, Ontario, Canada), used in conjunction with the Eyelink Toolbox for Matlab^28^. Gaze recordings were calibrated with a standard 9-point calibration procedure^29^, and gaze position was recorded binocularly at a sampling rate of 1000 Hz. Online fixation monitoring was based on the average of the recorded gaze positions from the two eyes. Noise artifacts were reduced using Eyelink software, which applied a heuristic filtering algorithm to the raw gaze position samples (see Stampe, 1993, for details). Gaze data were classified using the Eyelink algorithm into saccades and fixations using velocity and acceleration thresholds of 30°/s and 8000°/s^2^, respectively.

### Stimuli

Stimuli were presented on a gamma-corrected 27″ BenQ XL2720Z LCD monitor controlled by a Dell Optiplex 9020 desktop computer with a Quadro K420 graphics card. The experiment was programmed in Matlab (The Mathworks, Inc., Natick, MA) using the Psychophysics Toolbox Version 3^30–32^. Display resolution was set to 1920 × 1080 and the refresh rate to 120 Hz. Observers were seated at a viewing distance of 50 cm from the display, with head position stabilized using a chinrest. At this distance, the display subtended 67.2° horizontally and 38.1° vertically. The maximum display intensity was 141 cd/m^2^.

Natural scene images were selected from the LabelMe image database^26^. As the full database includes a much larger number of images than required for the present study, a subset was created from a benchmark dataset (http://groups.csail.mit.edu/vision/LabelMe/Benchmarks/spain/), originally used for training a classifier to recognize different object categories, using the 4053 images listed under test and training sets (training and test images were used the same way, with no distinction between these two categories). It consisted of a variety of mostly outdoor scenes (e.g., a mix of urban and rural scenes) taken from different countries around the world, and selected in part because they were heavily annotated (median percentage of annotated area in each image = 92.9%). To maintain uniformity in stimulus dimensions across trials, we discarded images that did not have an aspect ratio of 4:3, which resulted in a final set of 3494 images. Each image was scaled to 48° in width and 36.9° in height, and the root-mean-square (RMS) contrast of each image was adjusted to a value of 0.5. On each trial, we selected a random image, without replacement, from this set. Images were displayed on a uniform gray (69.2 cd/m^2^) background.

Targets were 2D Gaussian patches that were intended to be highly visible across a large range of background luminance values and textures. These patches were generated by first producing an image negative of the natural scene image ([255,255,255]-[R,G,B]) within a 3.25° × 3.25° square centered on the x- and y- coordinates of the selected patch location (see *Procedure*). The pixel locations within this square region were then randomly scrambled and the resulting values were superimposed on the original image using alpha blending, with the alpha values defined by a 2D Gaussian profile (s = 0.85°), ranging from fully transparent to fully opaque.

### Procedure

For the duration of each trial, observers were instructed to maintain fixation on a 0.4° diameter circle (white 0.035° outline and black fill) at the center of the display. As shown in Fig. 1A, at the beginning of each trial, observers were shown the scene image and fixation dot. Target presentation was withheld until the observer’s registered fixation location continuously fell within a 1.5° × 1.5° square region centered on the dot for a randomly selected interval from 500 to 1250 ms. Following this interval, the target patch was presented for 50 ms at one of 72 possible stimulus locations, defined by the combination of 3 eccentricities (5°, 7.5°, 10°) and 24 angular locations (0 to 360° in 15° steps). After the target patch was removed, observers were instructed to report the location of the center of the patch by adjusting a crosshair using a mouse (0.50° × 0.50° black fill, and 0.14° stroke width, surrounded by a 0.04° white outline), while maintaining fixation at the center of the display. The crosshair was visible during the response phase only, and its position was reset to the center of the display at the beginning of the response phase on each trial. During this response phase, if the observer’s gaze position fell outside the 1.5° square centered on the fixation dot for longer than 150 ms, observers were given feedback in the form of a buzzer tone. In these instances, the trial was immediately aborted and no response was recorded. In addition, observers were instructed to press the spacebar if they did not see the patch at all, and the program advanced to the next trial without recording a response. Responses were otherwise recorded when the observer clicked to indicate their response. No feedback was provided regarding response accuracy. Trials were separated by a 500 ms blank interval with a uniform mean luminance gray screen.

Observers first completed a short block of 45 practice trials of the task, followed by 720 trials of the main experiment, which consisted of 10 trials for each possible patch location, presented in a random order. Drift correction of the eye tracker was performed every 45 trials, at which time observers were also shown a screen indicating their progress (number of trials completed) through the experiment. In addition, the eye tracker was re-calibrated at least once every 180 trials.

The procedure for Experiment 2 was identical to that of Experiment 1, except observers were instructed to saccade to the target patch as soon as it appeared. After saccade onset, once the observer’s gaze position left the central 1.5° × 1.5° square region around fixation, a crosshair was displayed at the x- and y- coordinates of the currently registered gaze position from the eye tracker and remained on the screen for 500 ms following saccade onset. The marker was a crosshair similar to that used in Experiment 1 (black fill and a white outline, or a white fill with a black outline, randomly selected with equal probability). The purpose of the crosshair was twofold – to maintain participant alertness (as there were no other visual stimuli or task instructions), and to monitor tracking accuracy throughout the course of the experiment. Participants were instructed to notify the experimenter in the unlikely event that the crosshair appeared far from their point of gaze following the saccade. If this happened, the eye tracker was recalibrated at that point in the experiment. As in Experiment 1, drift correction and recalibration were also performed every 45 trials and 180 trials, respectively.

The procedures for Experiment 3 were similar to those for Experiment 1, with a change in the appearance of the natural scene images. Images were RGB color negatives ([255,255,255]-[R,G,B]) of the originals, and rotated 180° (i.e., flipped upside down) from the originals. As in Experiment 2, the contrast polarity of the crosshair in Experiment 3 was randomized (black outline and white fill, or the reverse). Observers reported the location of the target patch by adjusting the location of the crosshair using the mouse.

### Data analysis

Aborted trials due to breaks from fixation during the response interval accounted for 5.63% and 4.34% of all responses in Experiments 1 and 3, respectively. Aborted trials due to self-reported inability to see the patch accounted for 0.19% of responses in Experiment 1. As this represented a very small number of trials, this response option was removed in Experiments 2 and 3. To remove potential lapses, we additionally removed responses with an error magnitude (distance from the center of the patch) of more than 4 standard deviations from the mean (0.29%, 2.73%, and 0.30% of trials in each of the three experiments).

The purpose of the analysis was to determine the relationship between the direction of observers’ errors in the task and the features in the image. As shown in Fig. 1C, the influence of image features on localization errors was calculated by subtracting the image feature value (e.g., luminance) at the location of the physical center of the patch from the value at the coordinates of the observer’s response. These values were calculated from the original scene image without the target patch. For example, if localization were systematically biased toward dark areas in the image, we would see lower luminance values at the reported location compared to the physical center of the stimulus. Difference scores between these map values were calculated for each of the image statistics described below and then averaged across trials. In Experiments 1 and 3, the response coordinates were taken from the location of the mouse click. In Experiment 2, gaze data were classified into saccades and fixations using Eyelink software, with velocity and acceleration thresholds of 30°/s and 8000°/s^2^, respectively. To calculate difference scores, the coordinates were taken from the *x,y* saccade landing location of the first saccade (greater than 2.5°) following stimulus onset. Saccade landing coordinates were averaged across the left eye and right eye recordings. Across participants, the median saccade onset latency was 195.5 ms ± 10.4 ms (SEM). Figure S2 in the Supplemental Materials shows the distribution of saccade latencies in Experiment 2. In addition, Fig. S1 shows the distribution of response errors (distances of mouse clicks and saccades from the patch center) across the three experiments.

**Figure 2:**
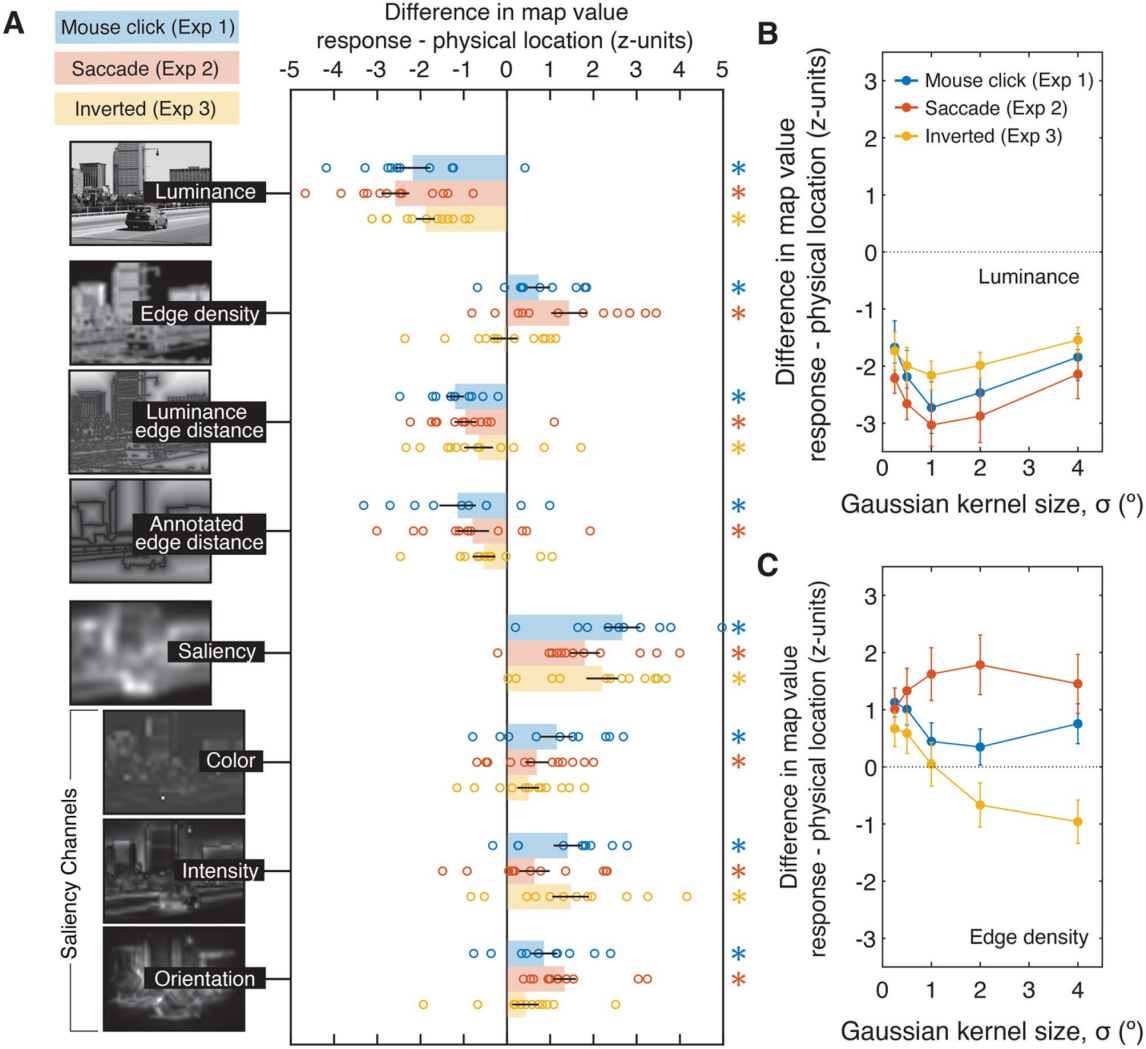
(**A**) Influence of image features on the direction of localization errors within natural images. Bars indicate the mean difference score for each image statistic (examples shown in the left column) in each experiment (Experiments 1, 2, and 3 shown blue, red, and yellow, respectively), with scatter points representing individual observers (see text for description of the direction of each effect). To facilitate comparison across the different measures, all values are expressed as the z-score of the observed mean difference score relative to the null distribution produced by the permutation procedure illustrated in Fig. 1B. Asterisks on the right side of the figure panel indicate significant differences from 0 based on individual permutation tests adjusted for a false discovery rate of 0.05 using the Benjamini–Hochberg procedure (see Supplemental Fig. S3B for histograms of the group null distributions). (**B**) Difference scores (in z-score units) for luminance, analyzed separately for each Gaussian kernel size (standard deviation, σ). (**C**) Difference scores for edge density at each kernel size. Error bars in each panel represent ± 1 standard error of the mean (SEM).

Image value difference scores were calculated for eight image statistics (including three individual saliency channels). These measures fell into three broad information categories: luminance, edges (edge density, distance from the nearest edge, distance from labeled object boundaries), and saliency. Figure 2A shows 2-D maps illustrating each measure for the same image. To measure the effect of luminance on observers’ responses, we calculated difference scores based on the intensity value after blurring the image using a 2-D Gaussian kernel at each of five different standard deviations (σ): 0.25°, 0.5°, 1°, 2°, and 4°.

Edge density was calculated by identifying the luminance edges in the image using the Canny edge detection algorithm, and then spatially smoothing the binary output using a Gaussian kernel with the same set of five standard deviations (0.25° through 4°). Higher values represent regions of the image with a greater density of edges. Edge distances were calculated by first extracting the luminance edges using the Canny algorithm, and then applying a Euclidean distance transform on the binary edge map to calculate the distance of each pixel from the nearest edge (i.e., higher values represent larger distances from the nearest edge). Similarly, distances to object boundaries were calculated from a binary map of all the outlines of the LabelMe annotations in the image. A Euclidean distance transform was applied to this map, producing a map of the distance of each pixel from the nearest edge.

Saliency was calculated using the procedures and parameters described in Itti, Koch, and Niebur^27^, with saliency computed from the sum of three normalized conspicuity maps, representing color, intensity, and orientation contrast. To summarize, the model consisted of seven channels: two for color (red-green and blue-yellow color differences), one for intensity, and four for orientation (0°, 45°, 90°, and 135°). For each channel, maps across different spatial scales were generated using dyadic Gaussian pyramids and center-surround difference maps were calculated from paired differences across these spatial scales. We note that, although there have been many refinements to the original saliency model, with the goal of predicting fixation patterns in free-viewing of natural images (e.g.,^33^), we selected this model as a simple way to summarize center-surround differences image content, and to quantify possible landmarks in the images.

Significance testing was performed using permutation tests (Fig. 1C), in which the observed values were compared to permuted null distributions, which account for general biases present across all images. For example, independent of image content, a subject might have a tendency to report locations that are below the target’s physical location, or positions toward the center of the image, which could coincide with changes in these image features. As shown in Fig. 1C, to control for these factors, each null distribution was calculated by randomly shuffling the relationship between the specific image and the response coordinates. For example, for the luminance difference score, the physical and reported patch locations on trial 1 might be used to calculate a difference score based on the luminance values on trial 7, and so on. This mapping was shuffled between trials and repeated for 1000 iterations to calculate a null distribution of differences. In order to report measurements of effect size on the same scale across the different measures, for each observer, we calculated a z-score, representing the number of standard deviations the observed value was from the center of the null distribution (see Fig. S3A in the Supplemental Materials), and these values are shown in Fig. 2. For each measure, a group null distribution was calculated by averaging the values in the null distribution across observers, and *p* values were calculated from the proportion of difference scores in the resulting null distribution that were more extreme than the mean observed difference score (see Fig. S3B). To correct for multiple comparisons, we ﻿controlled for a false discovery rate of 0.05 using the Benjamini–Hochberg procedure. Where difference scores were calculated for different spatial scales (luminance and edge density), we report difference scores averaged across all kernel sizes in Fig. 2A and separately for each spatial scale in Fig. 2B,C.

**Figure 3:**
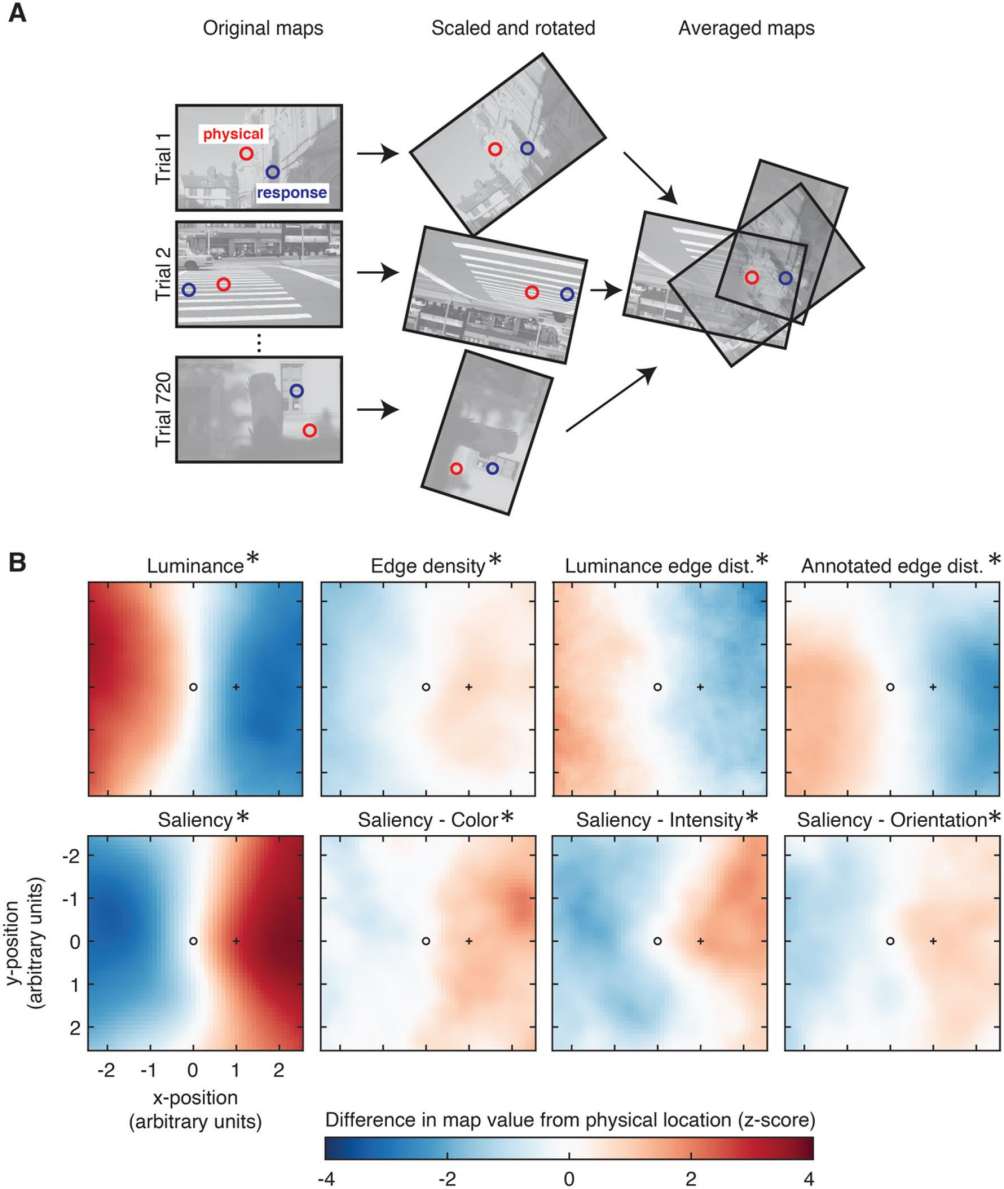
(**A**) Procedure for calculating averaged feature maps. Maps of each feature (in this example, luminance), were rotated and scaled to align the physical and response locations of each patch across all trials. The resulting maps were then averaged across trials, and across participants. (**B**) Averaged maps of each feature in Experiment 1. The coordinates x = 0, y = 0, indicated by the empty circle (○), correspond to the physical location of the patch, and the coordinates x = 1, y = 0, indicated by the plus symbol ( +) correspond to the location of the observer’s response. Following the procedures shown in Fig. 1C, values are shown as z-scores, calculated relative to a permuted null distribution of maps, where the SD for the calculation is the standard deviation of null distribution at the reported location. Asterisks indicate significant effects shown in Fig. 2.

In addition, to visualize local variation in image statistics near the physical and reported stimulus locations, maps of each image statistic were averaged across trials. To account for variation in error direction and magnitude across trials, as shown in Fig. 3A, images were rotated and scaled prior to averaging, such that physical and perceived locations were aligned across trials. As shown in Fig. 3B, the coordinates (x = 0,y = 0) and (x = 1,y = 0) correspond to the physical and response locations, respectively. As before, to facilitate comparisons between different measures, values are reported as the z-score of the observed difference relative to the permuted null distribution.

## Results

### Experiment 1: directional biases in observers’ localization errors

Figures S1 and S2 in the Supplemental Materials show the mean error magnitudes and latencies across the each of the three experiments. In each experiment, median localization errors (distance from the center of the patch ± SEM) were 1.08° ± 0.08°, 1.24° ± 0.07°, and 0.98° ± 0.05°, respectively. The primary goal of the analyses was to determine the influence of each image feature on these errors, by calculating a difference score based on the value of each feature at the physical stimulus location and the reported location. We isolated the image-specific effects using a procedure in which we compared the observed difference score to a permuted null distribution (Fig. 1C), produced by shuffling the mapping between images and their patch and response coordinates. This controlled for general biases (i.e., nonspecific effects) that might influence the difference scores—for example, a general downward bias^34^, or idiosyncratic errors^8^, which could coincide with changes in luminance, edge information, or saliency. The effects of any such biases would be present in the null distribution; comparing the observed difference score to this baseline controls for these non-specific effects.

Figure 2 shows the observed difference score relative to this null distribution, expressed as a z-score, corresponding to the number of standard deviations of observed value from the center of the null distribution, and *p* values were calculated from null distributions observed at the group level (see Fig. S3 in the Supplemental Materials for the individual distributions). In Experiment 1 (mouse click responses, shown in blue), these revealed significant effects related to luminance, edge information, and saliency. Specifically, observers’ responses were pulled toward the darker areas in the images, with negative values in the graph indicating lower luminance values at the response location compared to the physical location of the target patch (mean of individual observer z-scores: − 2.18, permutation test: *p* < 0.001). This analysis also revealed spatial tuning across different blur levels (Fig. 2B), with the largest effect observed with a Gaussian kernel with a standard deviation of 1° (mean z-score = -2.73). In other words, observers localized patches toward darker areas in the image, at a relatively coarse spatial scale, which may be a consequence of reduced acuity in the periphery. This is further supported by an additional analysis (Fig. S5 in the Supplemental Materials) which shows different spatial tuning for the patch eccentricity conditions. At 5° and 7.5° eccentricity, the largest effect was observed with a kernel size of 1°, while the largest effect at 10° eccentricity was observed with a kernel size of 4°.

In addition, we observed effects associated with edge information. For edge density, positive values in the graph indicate a bias toward areas of higher edge density (i.e., a higher edge density value at the response location compared to the physical location; z = 0.74, *p* = 0.003). As shown in Fig. 2B, this effect was largest at small kernel sizes. In addition, we observed significant effects of edge distance. Here, *negative* difference scores indicate lower edge distance values at the response location compared to the patch location, consistent with a bias *toward* edges. These values were significantly negative for edge distances based on both luminance-defined edges (z =  − 1.20; *p* < 0.001), and edges defined by annotated objects in the LabelMe database (z =  − 1.14; *p* < 0.001).

Finally, we observed significant effects of saliency, in which responses were pulled toward more salient regions of the image (z = 2.67). We tested whether this effect was driven by one saliency channel (color, intensity, or orientation) or whether these effects were seen across all channels. When the difference scores were calculated individually for each channel, in Experiment 1, we observed significant effects in each channel (color: z = 1.15, *p* = 0.001, intensity: z = 1.40, *p* < 0.001, orientation: z = 0.86, *p* = 0.006).

### Experiment 2: Changing the response modality

One possibility is that these biases may be a direct or indirect consequence of the response modality used to measure position judgments. For example, they may be influenced by observers’ preference for cursor placement (e.g., the cursor may be more visible on certain areas). In addition, with manual responses, observers’ reports are somewhat delayed from stimulus presentation. Even though observers were able to initiate their responses once the target disappeared, the time required to perform the task resulted in a mean response latency of 987.3 ms. This may introduce additional distortions related to the memory of the target’s location. To test whether such factors could account for the observed biases, we carried out a second experiment in which observers were instructed to saccade to the patch as quickly as possible, and we analyzed the landing coordinates of the saccade in place of observers’ mouse clicks. This reduced the possibility of observers introducing biases related to cursor adjustment, and greatly reduced the response latency (with a median saccade onset latency of 195.5 ms).

Here we observed very similar effects to those in Experiment 1 (Fig. 2; red bars). Observers’ responses were significantly biased toward darker areas (z =  − 2.58, *p* < 0.001), areas of high edge density (z = 1.44, *p* < 0.001), as well as toward luminance edges (z =  − 0.96, *p* = 0.001), and object boundaries (z =  − 0.79, *p* = 0.005). The pattern of tuning for edge density was different for saccades compared to mouse clicks, with the largest effect at observed with a Gaussian kernel with a standard deviation of 2° (Fig. 2B), but nevertheless, this effect was robust for a range of kernel sizes. As in Experiment 1, observers’ responses were also pulled toward more salient regions of the image (z = 1.80, *p* < 0.001). As in Experiment 1, these effects were significant when analyzed separately for each saliency channel (color: z = 0.69, *p* = 0.02, intensity: z = 0.64, *p* = 0.028, and orientation: z = 1.33, *p* < 0.001, respectively).

### Experiment 3: Inversion

One possibility is that these effects may depend on high-level scene processing. We tested whether these effects persist when scene processing is disrupted, by making the scene more difficult to recognize. Previous work has shown that inversion interferes with object recognition^35^ and high-level scene processing across a number of experimental paradigms^36–38^. In addition, scene categorization performance is reduced in abnormally-colored images^39^. The procedures in Experiment 3 were the same as Experiment 1, except images were color negatives of the originals that were presented upside-down (rotated 180°; see Fig. 1B). Using negatives also had the effect of changing the correlations between some of the image features (e.g., edge density and luminance; see *Correlations between features*). With these inverted images, we observed very similar effects of both luminance and saliency (Fig. 2; yellow bars), with responses pulled toward darker areas (z =  − 1.88, *p* < 0.001) and more salient areas of the image (z = 2.20; *p* < 0.001 for individual channels, color: z = 0.49, *p* = 0.10, intensity: z = 1.47, *p* < 0.001, and orientation: z = 0.43, *p* = 0.15). Although the sign of the difference scores for some of the measures associated with edge information stayed in the same direction as observed in the other two experiments, two of the effects were no longer significant (z =  − 0.07, and z =  − 0.53 for edge density and object distance, respectively, *p* values ≥ 0.069, while the effect of luminance edge distance was significant (z =  − 0.66, *p* = 0.02).

### Mapping feature influences

The main analysis in Fig. 2 shows the influence of local features using a two-point comparison based on the difference in feature values between the physical and reported locations of the patch. However, this only partially captures information about how these features influence position judgments within natural scene images. Do the changes in image statistics between the physical and reported locations of the patch follow a gradient (for example, uniformly decreasing luminance values between these two points), or are there more complex spatial patterns underlying these effects? For example, these effects could be driven by local minima/maxima of a given feature (e.g., small dark spot consistently surrounded by a light background), or there could be an interstitial feature (such as an edge) that appears consistently between the location of the patch and the response. Although such patterns are unlikely (as they would have to occur consistently across trials in order to produce these effects), we nevertheless created maps of feature influences for each effect shown in Fig. 3, to more clearly visualize our results.

To align images across trials, feature maps (e.g., luminance maps) were rotated and scaled such that the physical and reported locations of the target patch were co-registered across images, as shown in Fig. 3A. Figure 3B shows the final averaged maps for Experiment 1 (see Fig. S4 in the Supplemental Materials for the image maps for the other experiments), Within each panel, the coordinates x = 0, y = 0, indicated by the empty circle, correspond to the physical center of the patch, and x = 1, y = 0, indicated by the + symbol, correspond to the reported location. To facilitate comparisons with the bar graphs, values are expressed as z-scores relative to a permuted null distribution of mean feature maps, where the value at the physical location of the patch is fixed at zero, and the value shown at the reported location of the patch is the same as the values reported in Fig. 2. As shown in Fig. 3B, the spatial distribution of the image features around the physical and reported locations generally follows either an increasing or decreasing gradient. This indicates that the difference score based on the image values at these two points is generally sufficient to summarize the effects associated with each feature, and excludes the possibility of more unusual spatial patterns (e.g., small local minima/maxima, or interstitial features) underlying these effects.

### Correlations between features

**Figure 4:**
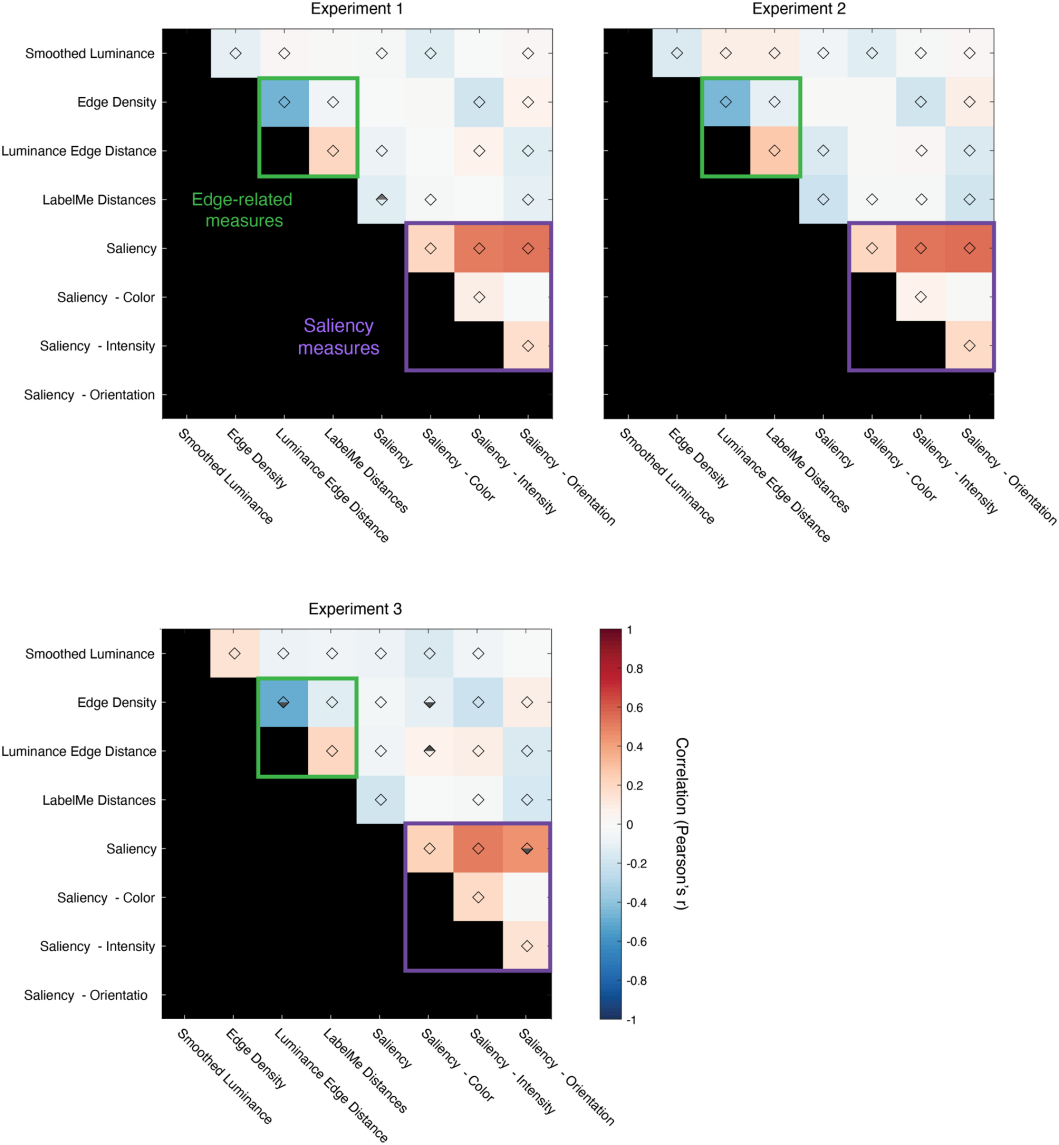
Correlation between image difference scores. Correlation values were calculated across all pairs of difference scores from all analyzed trials in each experiment (N = 6760, N = 8403, and N = 8239 in Experiments 1, 2, and 3, respectively). Diamond symbols indicate statistically significant correlations after controlling for a false discovery rate of 0.05 using the Benjamini–Hochberg procedure. Half-filled diamond symbols indicate correlations that are significantly above (upper-half filled) or below (lower-half filled) those in the permuted null distribution.

How much do these features capture redundant information about image content? We would expect a high degree of redundancy among the measures that are within the general categories tested (luminance, edges, saliency). For example, edge density and edge distance are derived from the same measure, and the individual saliency channels are necessarily associated with the overall measure of saliency. How much redundancy is there within these different categories, versus between them? Fig. 4 shows the correlations between the difference scores for individual trials for each of the measures shown in Fig. 2 (N = 6760, N = 8403, and N = 8239 in Experiments 1, 2, and 3, respectively). As expected, difference scores that were within these general categories (e.g., within the edge-related measures and within the saliency channels) were highly correlated. Overall, however, we observed relatively low, but statistically significant correlations across pairs of difference scores across all the measures used (mean of absolute Fisher z-transformed correlation values = 0.13, or R^2^ = 0.017). Are these internal correlations large enough to drive the patterns of results in Fig. 2? For example, edge density was significantly and negatively correlated with the intensity saliency channel (r =  − 0.19, *p* < 0.0001), even though the effects for these two measures go in the same direction (both positive z-scores) in Experiment 1. This indicates that these low correlations between image features may not be sufficient to account for all of the effects that we observed.

We therefore directly tested whether these correlations drive the biases we observed using two different approaches. First, we isolated effects that were specific to the observer’s responses within the images, using the same permutation procedure described earlier. In other words, we compared the values in Fig. 4 to those calculated from difference scores in the permuted null distribution, allowing us to identify effects that were specific to the images shown. We note that the correlations in the null distribution generally preserve the relationship between image features; in other words, features that were highly correlated within these images (e.g., correlations within the saliency channels) are also well-correlated in the null distribution. However, as shown in Fig. 4, we identified a small subset of correlation values (one pair of features in Experiment 1 and four in Experiment 3) where the observed correlation was significantly different from the null.

**Figure 5:**
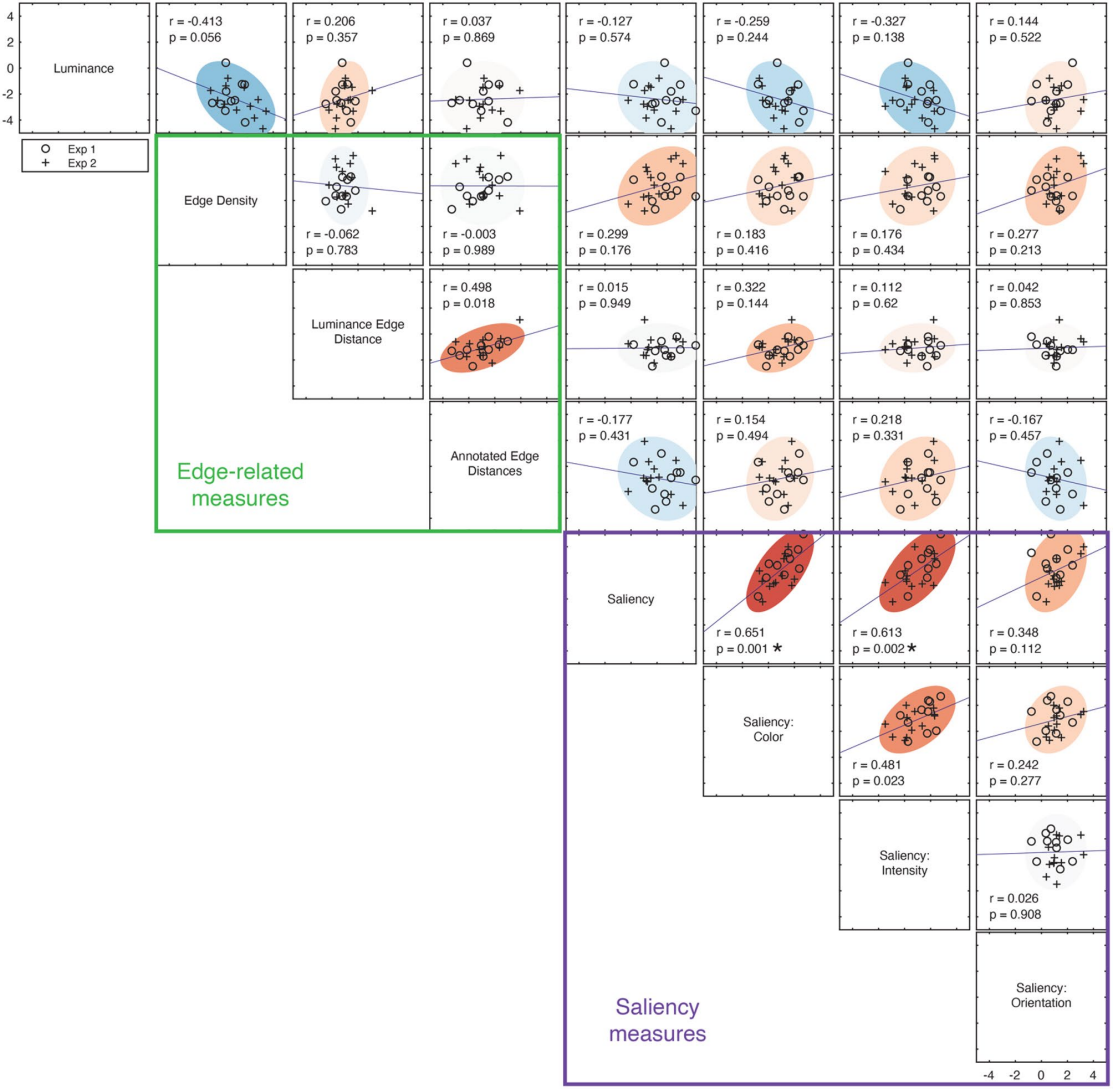
Between-participant correlations for all pairwise image features in Experiments 1 (N = 10) and 2 (N = 12). Each symbol represents the trial-averaged difference score (in z-units) for one participant in Experiments 1 (empty circle) and 2 (plus symbol). The correlations for Experiment 3, which used color negative images, are shown separately in Fig. 6. Filled oval regions represent 95% error ellipses, using the same color scale shown in Fig. 4. The asterisks (in two of the saliency-related measures) indicate a statistically significant correlation after controlling for a false discovery rate of 0.05 using the Benjamini–Hochberg procedure.

**Figure 6:**
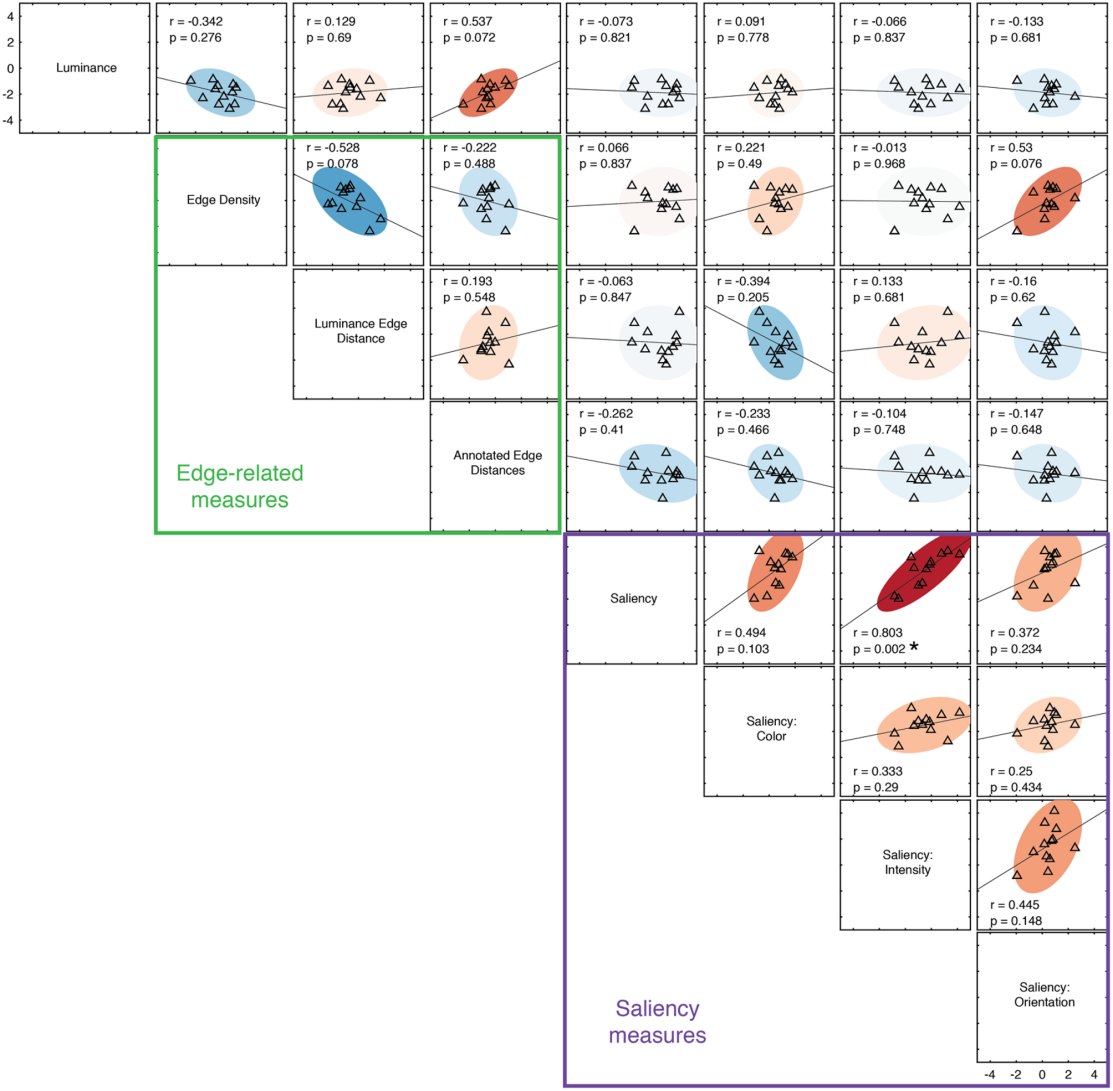
Between-participant correlations for all pairwise image features in Experiment 3 (N = 12). Each symbol represents the trial-averaged difference score (in z-units) for one participant. Filled oval regions represent 95% error ellipses, using the same color scale shown in Fig. 4. The asterisk (for the correlation between Saliency and Saliency: Intensity) indicates a statistically significant correlation after controlling for a false discovery rate of 0.05 using the Benjamini–Hochberg procedure.

Second, if the correlations between pairs of difference scores derived from different measures (Fig. 4) are largely responsible for the effects we see in Fig. 2, we would expect this to emerge at the individual observer level as well. In other words, for pairs of difference scores that are positively correlated, observers who exhibit a larger effect in one measure (e.g., luminance) should also show a larger effect in another (e.g., edge distance). Figures 5 and 6 show the same correlations between pairs of measures at the observer level. Across these analyses, at the individual participant level, only three of the correlations were significant (between saliency and two of saliency channels). In addition, a number of the correlations at the observer level were in the opposite direction from those calculated from the individual difference scores. Together, this suggests that the effects we observe are likely due to a constellation of different influences rather than exclusively driven by a single feature.

## Discussion

Our representation of an object’s location is heavily influenced by its visual context; however, much of the literature to date has examined these contextual influences with simple configurations and a limited number of objects. In contrast, this study measured the influence of local image features on observers’ position judgments of brief targets embedded within natural scene images, probing for these contextual effects across a number of perceptually relevant dimensions. The results of Experiment 1 indicated that observers’ responses were pulled toward darker image regions, as well as toward edges, and more salient regions of the images (Fig. 2). Critically, a follow-up experiment indicated that these errors were consistent across response modalities, even when responses occurred at very different time scales (987 ms on average for cursor adjustment versus 196 ms for saccades). In addition, a number of the reported effects persisted when the images were replaced with color negatives of the originals and physically inverted, manipulations which interfere with recognition^35,37–39^, and which changed the internal correlations between some of the image features (Fig. 4). Across all three experiments, we controlled for general directional biases in observers’ responses that coincide with changes in image features using a permutation-based analysis. This approach allows us to account for non-specific effects (e.g., response biases towards the center of the image, or towards the ground) and isolate those that are particular to the images seen on each trial.

Collectively, these results point to gravitational effects within natural scenes that are analogous to those observed with more simple configurations. Importantly, the gravitational landmarks are sources of information in the image—as we observed in each experiment, observers’ responses were generally biased away from sparse or featureless regions of the images, and toward edges or other informative content in the scene. In describing these effects, we generally refer to them as pull ‘toward’ rather than ‘away’ from specific features (e.g., a pull toward dark areas), to match the language used in other research describing landmark or anchor effects. However, we note that in principle, it could be described as biases away from other features (e.g., a pull away from bright areas). One possibility is that this constellation of effects may reflect priors for where objects are likely to be located within a scene. In many everyday tasks, observers need to make decisions regarding object location under conditions of noise or uncertainty, and these decisions could reflect the underlying assumption that a brief target would most likely be physically near other objects in the environment. The effects that we observed may point to some of the perceptually-relevant features that underlie these assumptions.

Although the effects of edges and saliency are largely consistent with an explanation based on local landmarks, the effects associated with luminance are more surprising. One possible account is that this may reflect a general assumption that darker regions are also associated with objects rather than empty space. However, this tendency for observers to report that the target was located on a darker portion of the image persisted when color negatives were used. Here, portions of the image that were previously higher luminance were now lower luminance in this experiment, indicating that this bias was independent of the identity of the objects in the image. Another possible explanation for this error pattern is that it may be related to previously reported black-white asymmetries seen in a number of different visual processes, including detection, motion perception, and grouping and object perception^40–42^, which generally show that luminance decrements are processed more effectively than increments. In addition, we note that there are other image-related factors, such as our use of a linearized monitor, as well as specific photographic attributes (e.g., exposure, dynamic range), that could have enhanced the subjective saliency of dark regions and contributed to the observed bias. Differentiating between these possible accounts would require further investigation.

Although we observed some of these effects (e.g., luminance) consistently across all experiments, not all effects survived the image manipulations in Experiment 3, in which the scene photographs were inverted and color-negatives of the originals. Specifically, gravitational biases toward two of the edge-related measures (edge density and annotated edge distance) were not observed in Experiment 3. One possibility is that these effects may depend on high-level object recognition, and therefore may be more likely to be disrupted by the inversion and color manipulations, particularly if identifying the outline of an annotated object is linked to recognition. Further work comparing the sizes of these effects within the same observers will be important to establish whether disrupting object recognition significantly changes the gravitational biases we observed.

Taken together, these results also complement previous findings in the literature, showing landmark effects in pointing or cursor movements. As we discuss, when reproducing the locations of previously seen targets, observers’ responses are pulled toward nearby landmarks or anchors, although more complex patterns can emerge when multiple landmarks are present^13,43^. This landmark effect in pointing or cursor responses also resembles another common observation in saccades—the global effect, or the tendency for saccades endpoints to fall near the center of mass when multiple objects are present^44–46^. Here, we observed that saccade landing locations were directed to the same set of features as observers’ cursor responses, toward edges or other informative content in the scene, which could be a result of averaging or assimilation between the target and these features in the environment. However, previous work has also shown that the global effect varies with the timing of the target and distractor, with no global effect observed for saccades to a brief target when the distractor is continuously visible prior to target onset^47^, as was the case in our experiments. It is therefore possible that the saccade errors observed here reflect different mechanisms than the global effect in saccades.

In addition, our results demonstrating that saccade landing positions are directed to more salient regions of the image are broadly consistent with saliency models to predict eye movements during free-viewing or more naturalistic tasks^27,33^, however this work has not generally examined the relationship between saliency and error in reflexive saccades to unrelated targets. We also note that we observed effects of saliency on observers’ responses across both short and long time scales (i.e., saccades and mouse clicks), whereas previous work has shown that the effects of saliency on eye movements are larger at shorter timescales^48–50^. However, in all our experiments, observers previewed the scene while maintaining central fixation for 500–1250 ms prior to patch onset (Fig. 1A), and it is possible that removing or shortening this initial fixation in future work might reveal variation in the effect of saliency or other visual features on observers’ responses at different time scales.

Another related line of work has used similar approaches to identify category-based biases in reporting the remembered location of an object. Specifically, the category adjustment model of spatial memory^12^ posits that memory-based reports of an object’s spatial location are weighted by categorical information, where position judgments are biased toward central, or prototypical locations (e.g., the center of a quadrant within a circle). Holden et al.^51^ demonstrated that this model can be applied to natural scene images. When reporting the locations of a target dot previously shown within a scene image in a delayed response task (with an intervening image shown during retention), observers’ errors were pulled toward the center of color-defined clusters in the images. Unlike the present study, these effects were altered when inverted and color negative images were used, suggesting that those effects may depend on higher-level processes related to the extraction of semantic information from those images. More recently, using a serial reproduction technique, Langlois et al.^52^ demonstrate that memory-based spatial judgments are pulled toward regions in natural images where position discrimination accuracy is high. While it remains to be seen to what extent memory is involved here, we note that we observed similar effects for immediate saccades, which were on a very rapid timescale, and where any influence of memory is likely to be minimal. As the targets here were intentionally selected to be brief and diffuse to produce a high degree of uncertainty in position judgments, these responses necessarily occurred following stimulus disappearance. Regardless of whether memory is involved, the effects we observed occurred for immediate judgments of spatial location, which are highly relevant for localization in more naturalistic settings.

While these results indicate an assimilation or gravitational effect in natural scene images similar to those observed in more simple configurations, one possible interpretation is that biases may be an artifact of poor visibility of the targets on some of the backgrounds we used. The procedure for creating the target patches (using color negatives and scrambling the pixels) was intended to produce high visibility across a range of backgrounds, and the very low percentage of trials in which observers indicated not seeing the patch (less than 1% of trials) suggests that this was the case. Nevertheless, it is possible that for a given target patch, different portions may be more or less visible (e.g., a patch that straddles the boundary between two different surfaces in the image) which would bias the apparent location of its center. However, this account mostly generates predictions *opposite* to the effects observed in the present experiments. Targets would be expected to be less visible or identifiable in areas where the density of local features is higher^21,22^. For example, a target patch positioned at the boundary of a densely-textured portion of the image and a relatively sparse region would be more visible in the sparely-textured region, biasing position judgments *away* from areas where it is less visible. These effects are therefore unlikely to be a result of visibility-related stimulus artifacts.

Together, these results identify features within natural scene images that influence observers’ immediate reports of spatial location. Analogous to classic position landmark or assimilation effects, observers’ responses were pulled toward salient or informative landmarks within natural scene images, in a manner that was image-specific and independent of response modality, and robust to manipulations that interfered with identification, but preserved the spatial structure of the scene. While we observed these effects for immediate responses, further work will be required to determine the extent to which these relate to category-based effects in spatial memory, or priors for the distribution of objects within natural scenes. Another remaining question is the extent to which these effects depend on the identity of the target. Although the targets used in this study were defined by low-level features, using annotated objects as targets in future studies would allow examination of how identity-specific priors for target location might interact with scene information to influence position judgments.

## Supplementary Information

Supplementary Information.

## Data Availability

All data and materials for the study are available on the Open Science Framework online at: https://osf.io/d7qrv/.
