# Supplementary material for: Gravitational effects of scene information in object localization
Source: Sci Rep. 2021 Jun 1;11:11520. doi: 10.1038/s41598-021-91006-8 (PMC8169838; doi:10.1038/s41598-021-91006-8)
Supplement: Supplementary file 1 — Supplementary Information. [file 41598_2021_91006_MOESM1_ESM.docx]

**Supplemental Materials:** Gravitational effects of scene information in object localization

Anna Kosovicheva, Peter J. Bex


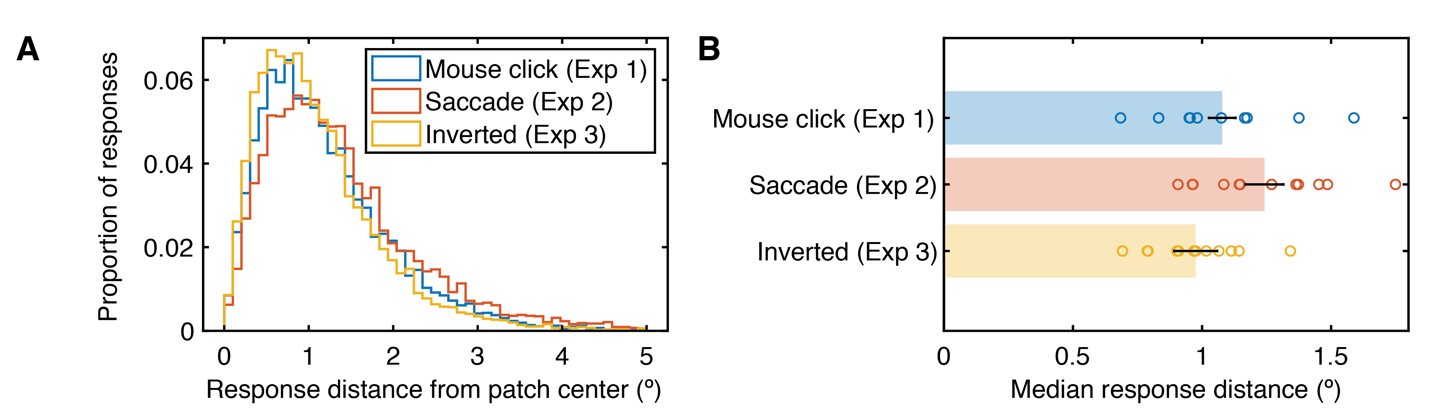


**Figure S1.** Distribution of response errors (i.e., the distance of the mouse clicks or saccade landing locations from the center of the patch) across observers in Experiments 1 through 3. (A) Histograms of all responses in each experiment (Experiments 1, 2, and 3 shown blue, red, and yellow, respectively). (B) Median response errors in each experiment were 1.08º, 1.24º, and 0.98º. Scatter points represent individual observers, and error bars represent ± 1 SEM.


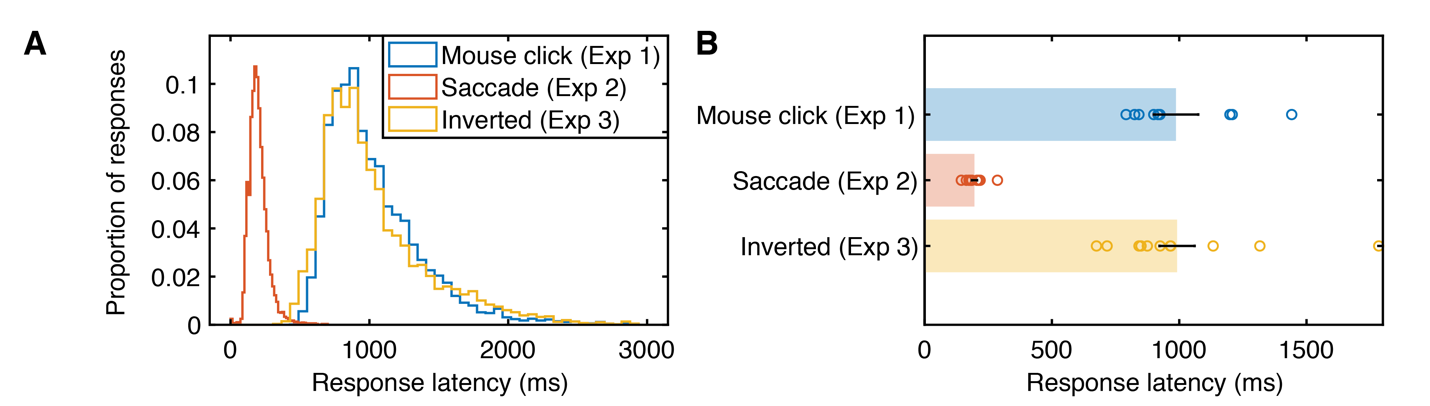


**Figure S2.** Distribution of mouse click latencies sand saccade onset latencies in Experiments 1 through 3. (A) Histograms showing the distribution of latencies for all responses in each experiment. (B) Median onset latencies in each experiment were 987, 196, and 992 ms. Scatter points represent individual observers, and error bars represent ± 1 SEM.


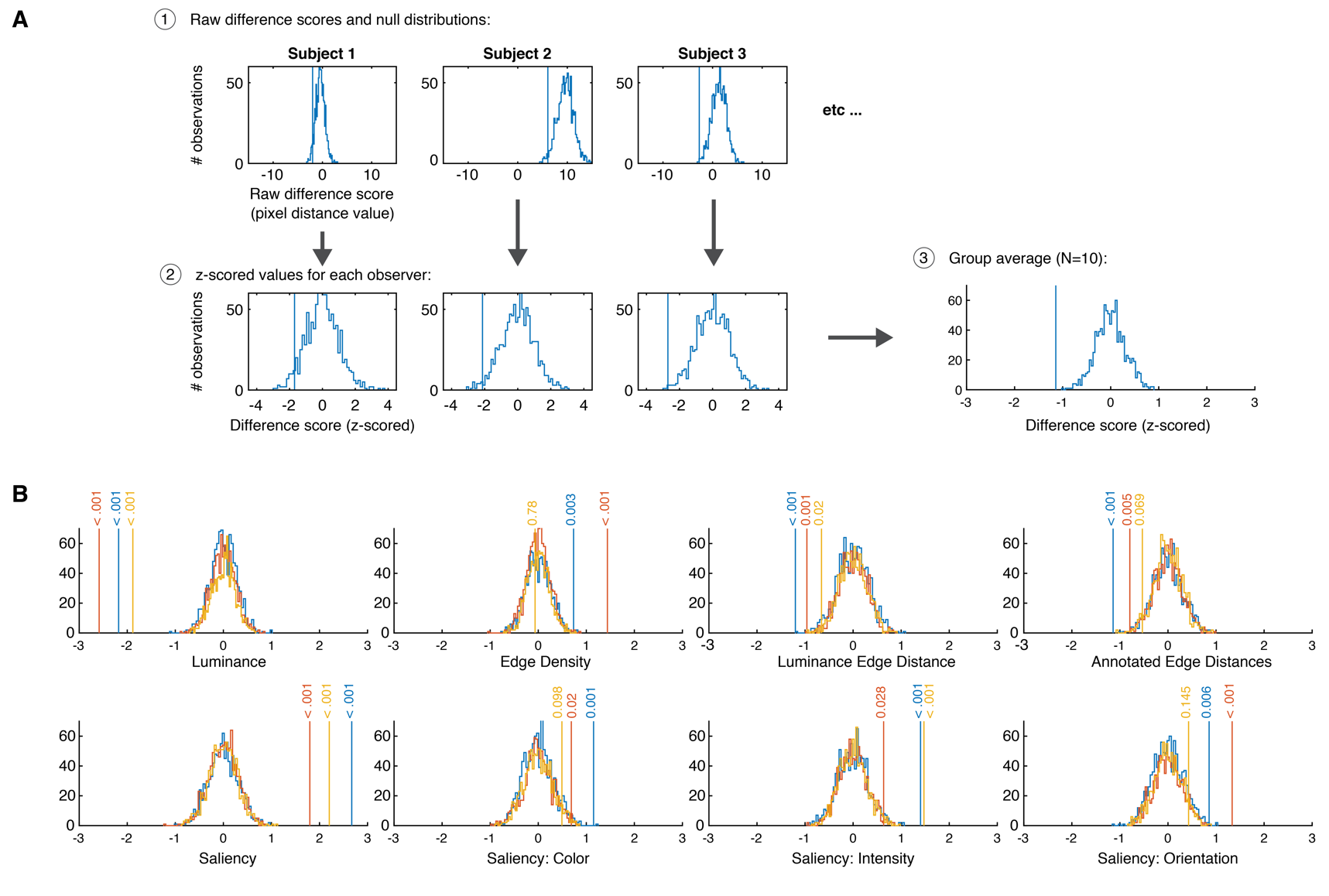


**Figure S3.** (A) Diagram outlining the procedure for permutation tests for each image statistic. (i) For each participant and each statistic, a null distribution was generated by shuffling coordinates following the procedures described in the Methods and Figure 1C. Examples are shown here for three observers in Experiment 1, showing luminance edge distance values in pixel units. Note that the mean and SD of the resulting null distributions varied across participants. (ii) To facilitate comparisons across measures and subjects, the values in each distribution was converted to z-scores, and the scatter points in Figure 2A indicate the z-score of the observed difference score for each participant. (iii) To calculate statistics at the group level, these z-scored null distributions and difference scores were averaged across participants (shown here for the ten observers in Experiment 1). (B) Group-averaged null distributions for each image feature and each experiment (Experiments 1, 2, and 3 in blue, red, and yellow, respectively), with colored vertical lines indicating the group averaged difference score. For each statistic, p-values were calculated from the proportion of observations in the null distribution that were more extreme than the observed value.


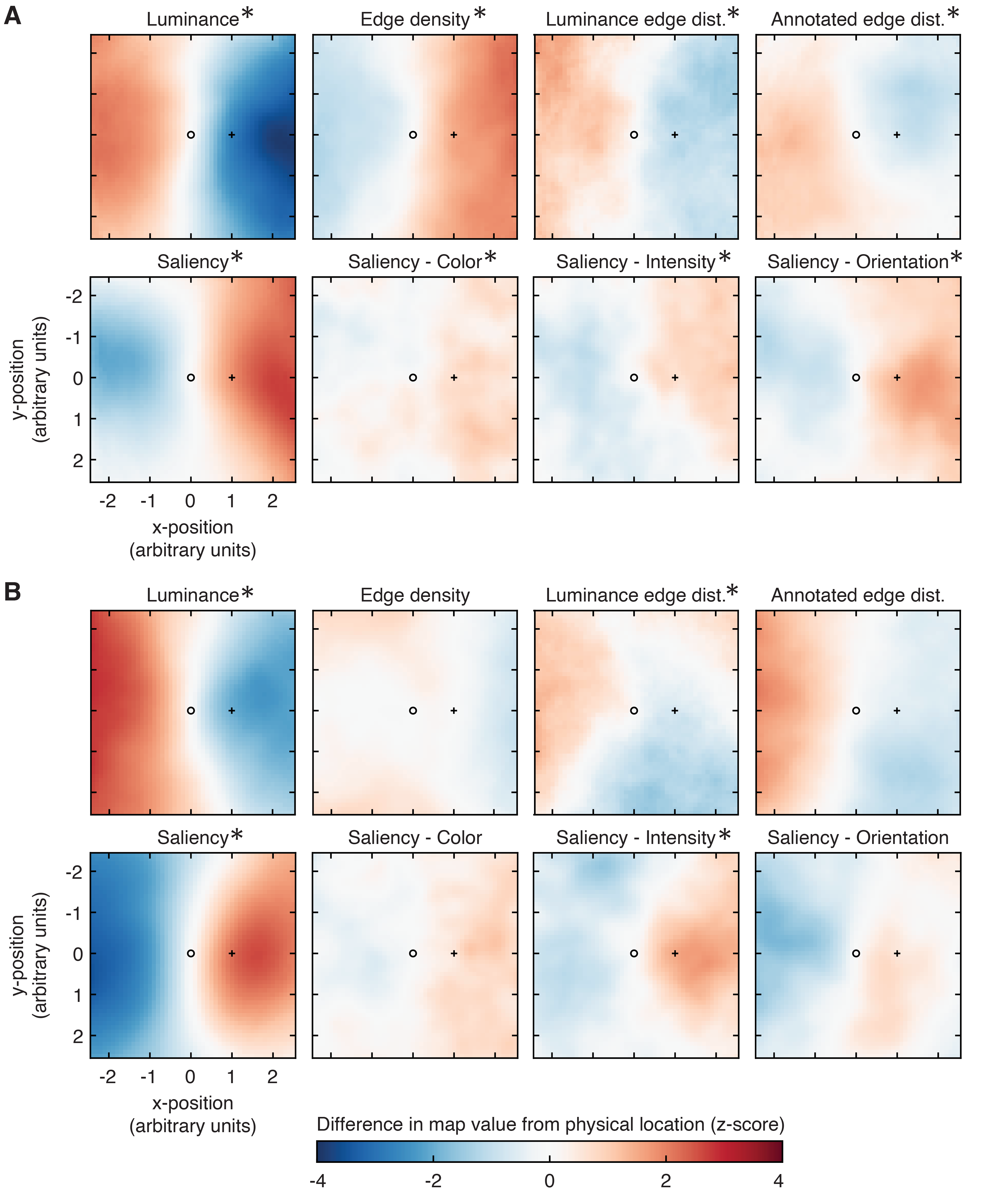


**Figure S4.** Averaged maps of each feature in (A) Experiment 2 and (B) Experiment 3, following the same procedures illustrated in Figure 3. The coordinates x=0, y=0, indicated by the empty circle (○), correspond to the physical location of the patch, and the coordinates x=1, y=0, indicated by the plus symbol (+) correspond to the location of the observer’s response. Values are shown as z-scores, calculated relative to a permuted null distribution of maps, where the SD for the calculation is the standard deviation of null distribution at the reported location. Asterisks indicate significant effects in shown in Figure 2.

**
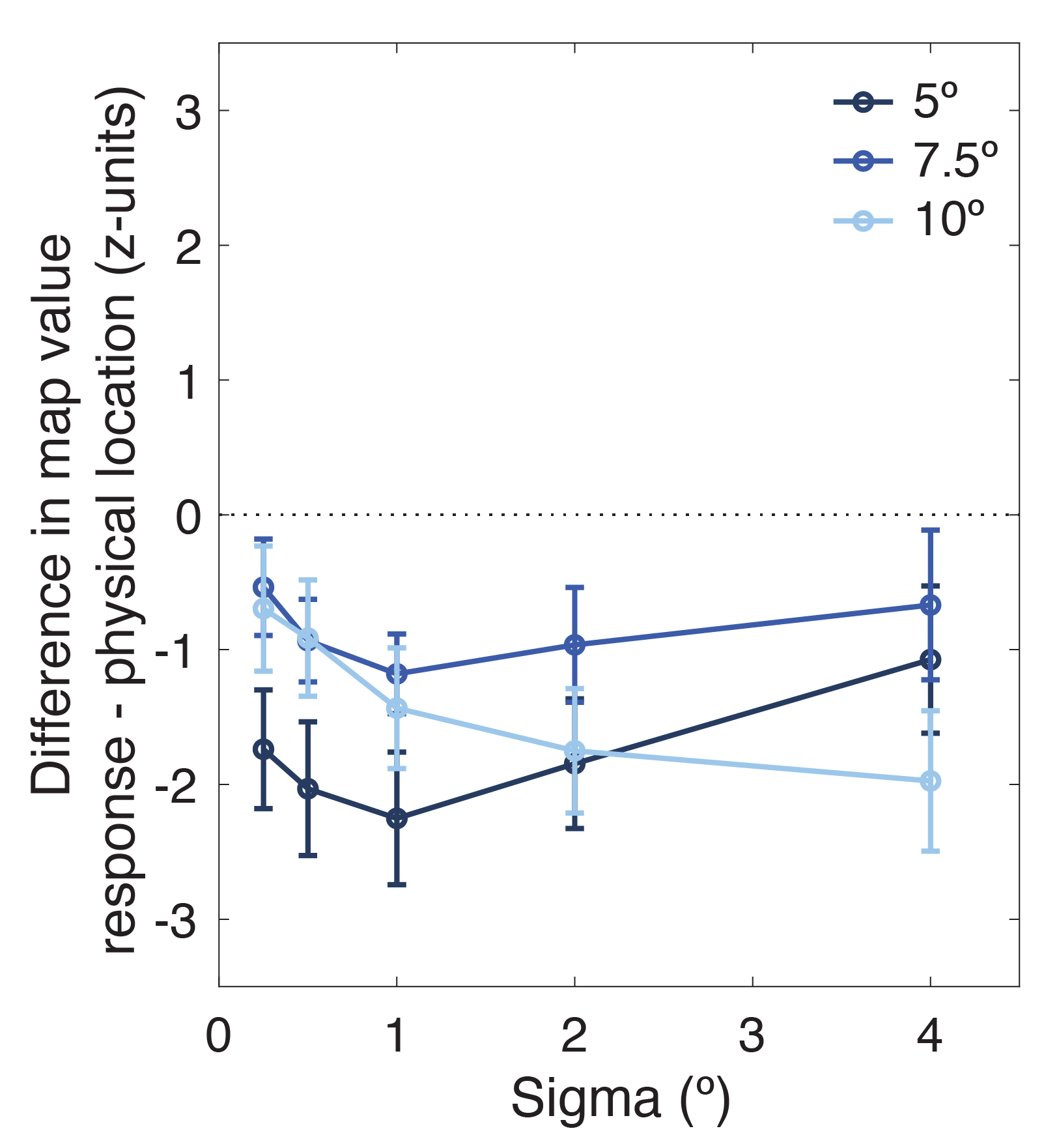
**

**Figure S5.** Difference scores (in z-score units) for luminance, analyzed separately for each Gaussian kernel size (standard deviation, σ) and patch eccentricity condition. Note that z-scores are not directly comparable to Figure 2, as here they were calculated separately for permuted null distributions calculated *within* each eccentricity condition. At 5º and 7.5º eccentricity, the largest effect was observed with a kernel size of 1º, while the largest effect at 10º eccentricity was observed with a kernel size of 4º. Error bars represent ± 1 standard error of the mean (SEM).
